# Perception of Leitmotives in Richard Wagner's Der Ring des Nibelungen

**DOI:** 10.3389/fpsyg.2017.00662

**Published:** 2017-05-04

**Authors:** David J. Baker, Daniel Müllensiefen

**Affiliations:** ^1^Music Cognition and Computation Lab, School of Music and Dramatic Arts, Louisiana State UniversityBaton Rouge, LA, USA; ^2^Music, Mind and Brain Lab, Department of Psychology, Goldsmiths, University of LondonLondon, UK

**Keywords:** musical memory, leitmotives, opera, symbolic notation, computational Modeling

## Abstract

The music of Richard Wagner tends to generate very diverse judgments indicative of the complex relationship between listeners and the sophisticated musical structures in Wagner's music. This paper presents findings from two listening experiments using the music from Wagner's *Der Ring des Nibelungen* that explores musical as well as individual listener parameters to better understand how listeners are able to hear leitmotives, a compositional device closely associated with Wagner's music. Results confirm findings from a previous experiment showing that specific expertise with Wagner's music can account for a greater portion of the variance in an individual's ability to recognize and remember musical material compared to measures of generic musical training. Results also explore how acoustical distance of the leitmotives affects memory recognition using a chroma similarity measure. In addition, we show how characteristics of the compositional structure of the leitmotives contributes to their salience and memorability. A final model is then presented that accounts for the aforementioned individual differences factors, as well as parameters of musical surface and structure. Our results suggest that that future work in music perception may consider both individual differences variables beyond musical training, as well as symbolic features and audio commonly used in music information retrieval in order to build robust models of musical perception and cognition.

## 1. Introduction

While Richard Wagner and his music have been the topic of a wide range of musicological and music theoretic research (Bailey, 1977; Deathridge and Dahlhaus, 1984; Dreyfus, 2012), the compositional techniques Wagner developed and their effect on listeners has not received nearly as much attention from the music psychology community. This may be due to the fact that Wagner's music does not make use of tonality in the traditional sense, but rather has been aptly described by David Huron as “contracadential” and very harmonically sophisticated (Huron, 2006). Huron notes that the complexity in Wagner's music may be attributed to its cadential content in that his cadences are “almost entirely divorced from perceptual or formal segmentation” (Huron, 2006, p. 338) making his music difficult to process for listeners who do not have prior listening experience.

In addition to the difficulty delineating cadential structures in his music, Wagner also composed his melodic material in order to avoid the regularity that is found in other 19th century composers (Dahlhaus, 1980; Grey, 2007). This conscious choice to write melodic material that seems to be endless and avoids easy segmentation often leads to difficulties for listeners, which results in thwarted and delayed expectations of musical events. Despite these inherent difficulties in parsing his cadential and melodic material, the continued popularity of his music for people at various points in history (Magee, 1988) seems to suggest that listeners from a wide range of backgrounds are able to process and enjoy the complex auditory scenes in his music.

Initial work investigating how listeners are able to hear salient musical material in Wagner's music was carried out by Deliège (1992) in order to demonstrate the principles of musical cue abstraction (Deliège and Mélen, 1997). Cue abstraction is rooted in Gestalt schematization processes inspired by the work of Lerdahl and Jackendoff (1983) and uses grouping and similarity-difference principles in order to predict where listeners will perceive musical boundaries as well as salient musical events. Deliège's studies on the perception of Wagner's music focused primarily on leitmotives, which are short musical ideas that can be used to refer to people, places, or ideas related to the musical narrative (Hacohen and Wagner, 1997).

**Figure 1:**
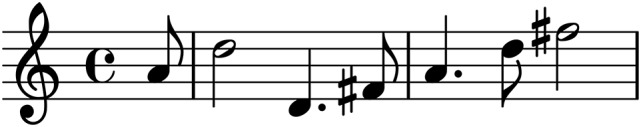
**The ***Schwert-Motiv*** in D major**.

**Figure 2:**
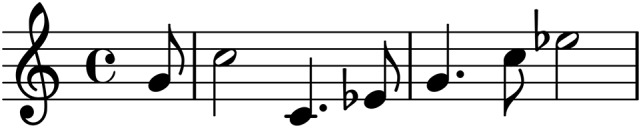
**The ***Schwert-Motiv*** in C minor**.

Leitmotives are ideal cues for studying salient musical events because they can exist in a multitude of permutations that are all perceived as the same cognitive entity. For example, the *Schwert-Motiv*, while often played in the major mode on the trumpet, can also be orchestrated with varying mode, range, and timbre in order to successfully convey the correct musical emotion the composer intended. Despite these changes, the leitmotive is often recognized as the same categorical entity as demonstrated in Figures 1, 2.

Using leitmotives as cues, Deliège demonstrated her cue abstraction principles, which model real-time music listening, were able to accurately predict salient musical events in non-tonal music (Deliège, 1992). Her initial findings showed higher leitmotive recognition rates in participants with more musical training, indicating that listener background played a significant role in the identification of salient musical events. Deliège has also demonstrated the success of the cue abstraction mechanism with the music of Bach (Deliège, 1996), Berio (Deliège and El Ahnmadi, 1990), and Boulez (Deliège, 1989).

Morimoto, Kamekawa, and Marui extended research on leitmotives by investigating the effect of extra-musical verbal information on the memorization and recognition of leitmotives. They found that exposing listeners to different types of verbal information in relation to musical material and the narrative did not result in any significant differences in the ability to recognize and memorize leitmotives (Morimoto et al., 2009). In a similar way, Albrecht and Frieler (2014) investigated how additional visual information (i.e., the events on the opera stage) might contribute to an individual's leitmotive recognition rate. They found that seeing and hearing the opera actually decreased an individual's ability to identify leitmotives in the auditory signal and hence suggests that visual information can act as a distractor in terms of encoding leitmotives.

Similar to much of existing work in music psychology, these previously mentioned studies investigating the perception of leitmotives categorized listeners based on their previous musical training. While musical training has been shown to be a factor that can contribute to performance in both tasks of perception (Besson et al., 2007; Williamson et al., 2010) and discrimination (Vuust et al., 2005) when investigating individual differences on musicality, much of this research unsystematically classifies participants into binary categories (such as “musicians” and “non-musicians”), primarily considering their years of formal musical training on an instrument as an indicator of their musical skills and experience. This somewhat arbitrary divide fails to consider other types of musical engagement or abilities other than instrumental skills (e.g., different types of perceptual abilities) which can also be deemed central to an individual's musicality (Levitin, 2012) or musical sophistication (Müllensiefen et al., 2014).

There is a lot of evidence from recent empirical research showing that scaled (i.e., continuous or ordinal as opposed to categorical or binary) measures of musical skills and experience represent good predictors in models of music perception and cognition (Chin and Rickard, 2012; Schaal et al., 2015; Bouwer et al., 2016), especially when considering musical background in the general population. While the aforementioned studies tend to reflect differences measuring individual's musical training, other studies have suggested that factors outside of musical training such as familiarity with the genre or style of the musical material (Tervaniemi, 2009; Hansen et al., 2016) as well as other non-performative abilities can play a crucial role in perceptual models (Bigand and Poulin-Charronnat, 2006). Though literature is sparse regarding perceptual models that takes into account genre familiarity, there are a number of studies that aim at mid-level features, such as schematic expectations (Eerola et al., 2009), and that do take into account listener backgrounds and musical acculturation that can be integrated in the modeling process via mechanisms such as statistical learning (Krumhansl et al., 2000).

We hypothesized that it might be possible to measure a listener's previous exposure to the music of Richard Wagner and use that measure as a predictor for their ability to recognize and remember cues in Wager's music. A previous study by Müllensiefen et al. (2016) found evidence that an individual's knowledge of and affinity for Wagner's music predicts memory accuracy for leitmotives in an experimental setting. In this particular experiment, expertise for Wagner's music was a stronger predictor than the amount of musical training for the participants' performance in the melodic memory experiment. These results suggested that an individual's prior exposure and understanding of a genre, and in particular Wagner's music, does in fact play a significant role in the understanding of complex musical passages and the extraction of and memory for leitmotives.

In addition to individual differences between listeners in terms of general musical expertise and familiarity with Wagner's music in particular, features of the musical material itself are certainly also responsible for the degree to which the cognitive decoding of Wagner's music can be successful. Numerous studies from 1970s onwards have demonstrated how structural features of music can facilitate or hinder cognitive processing (Dowling, 1971, 1972; Dowling and Fujitani, 1971; Cuddy et al., 1979). However, much of this work made use of artificially constructed musical stimuli with the primary aim to control the features of the musical material used in the experimental setup, but sometimes at the expense of the ecological validity and generalizability to real music.

More recent work from music informatics and systematic musicology has suggested computational measures that produce feature descriptions of real music excerpts in symbolic encoding that can be used successfully in models of music perception and cognition (Pearce and Wiggins, 2006; Müllensiefen and Halpern, 2014; Collins et al., 2015; Vempala and Russo, 2015; Wiggins and Forth, 2015) Hence, one aim of this study is to employ computational measures of musical structure with leitmotives from Wagner's music and assess to what degree they are predictive of cognitive behavior. Complimentary to structural features of leitmotives via symbolic encoding, we also aim to assess how the similarity in sound between individual leitmotive excerpts and their occurrence in a musical context contributes to perceptual and cognitive decoding. There is a growing body of research demonstrating the usefulness of sound and audio features developed within the music information retrieval (MIR) framework for describing the development of general preferences and taste over time (Serra et al., 2013; Mauch et al., 2015), cognitive attributes like the catchiness of pop songs (Burgoyne et al., 2013; Van Balen, 2016), or perceived emotional content (Friberg et al., 2014). Specifically, in this study we assess similarity by comparing chromagram data derived from audio excerpts (Müller and Wapnewski, 1992; Mauch et al., 2015).

In summary, this study intends to assess how features of the compositional structure and audio similarity on one hand, as well as individual musical sophistication and expertise with regard to Wagner's music on the other affect recognition memory for leitmotives. Thus, the study aims to combine predictors reflecting features of the musical material and traits of the listener in a single model of perception and memory for leitmotives in Wagner's music. Specifically, we hypothesize that knowledge and affinity for Wagner's music music can be interpreted as proxies for familiarity with his leitmotive technique and should therefore have positive effects on leitmotive processing and memory. In addition, general musical training should also aid processing and memory on the experimental task, consistent with findings from previous experiments (Dowling, 1978, 1986; Harrison et al., 2016). The ability to speak German may also provide a processing advantage in this experiment since the German vocals might provide extra clues toward the decoding of leitmotives and musical events in the auditory scene. Wagner's leitmotives are often paired with certain terms or ideas from the libretto that we believe could enable participants who speak German to encode the musical structure of the leitmotives together with semantic connotations. This ability to bind multiple features and aspects of an a object at the encoding stage could then support retrieval processes in the recognition task. This assumption is in line with evidence from experimental studies that have shown a similar differential memory advantage of presenting music and text together (Serafine et al., 1984, 1986). Accounting for German speaking abilities was also incorporated into the design in order to account for any German text that could have been recognized in the exposure phase since recordings of live opera were used.

In terms of features of structural complexity we expect more complex leitmotives to be processed and remembered worse (Harrison et al., 2016). Finally, we hypothesize that the similarity in terms of sound (i.e., audio features) between an individual leitmotive and any similar sound but not identical parts in a longer passage can act as distractor and hence decrease memory performance.

We employ a cross-over experimental design that makes use of two scenes from Wagner's *Ring des Nibelungen*. The design allows us to use leitmotives that were the lures in the memory test of Experiment I to serve as correct responses in Experiment II and vice versa. Thus, the findings from both experiments can potentially replicate each other and therefore the design helps to disentangle incidental features of the leitmotives used as experimental stimuli from the parameters of interest (i.e., compositional structure and audio similarity) that should have the same effect in both experiments.

## 2. Methods

### 2.1. Overall design

This study consisted of two experiments that used the identical experimental design and procedures: In both experiments an approximately 10 min scene was played to participants followed by a surprise memory test for 20 leitmotives, some of which were present in the scene previously played (*old* items) and others that had not been present in the scene (*new* items). The two experiments were set up to replicate the findings from each other and thus reduce the effects of incidental features and hence ensure a greater robustness of the overall findings. The 10 *new* items in Experiment I were used as *old* items in Experiment II and the 7 *old* items in Experiment I were *new* items in Experiment II. The passages used were picked for their overlap in musical material, but due to using ecologically valid stimuli an even split on leitmotives was not possible.

### 2.2. Overall procedure

Participants were tested in small groups. Upon arriving at the experiment participants signed a consent form and received the experimental instructions, which instructed them to listen attentively to a 10 min passage from a live recording of *Der Ring des Nibelungen* and subsequently answer some questions about the music. Participants listened to music via a pair of stereo speakers sitting at distances from 1 to 4 m from the speakers and via an initial sound check it was confirmed that the volume of the audio was set to a comfortable level for all participants. After the exposure phase participants were handed a response sheet and started the test phase. Here, participants were played 20 short leitmotives for each of which they had to indicate the perceived pleasantness of the leitmotive on a 7-point scale, a binary indication (yes/no), whether this particular leitmotive occurred in the 10 min passage from the exposure phase, and a corresponding confidence rating on a 7-point scale. In addition, they also rated valence and arousal expressed by the leitmotive based on the Russell's circumplex circle of emotion (Russell, 1980). Questions were set up on their response sheet in the the order listed above and participants were asked to fill out the questions in order. Shorter leitmotives were repeated up to 3 times with a 3 s pause between repetitions, such that each leitmotive item in the test phase lasted approximately 20 s and was followed by a silent gap of 10 s before the next leitmotive was played. In total participants had approximately 30 s to make all five ratings (pleasantness, explicit memory, confidence, valence, arousal) and were told to complete their ratings before the next leitmotive was played. The order of leitmotives was randomized across two different lists to mitigate any order effects. Following the test phase participants completed a set of questionnaires assessing their musical background and Wagner affinity and expertise. Ethical approval was obtained from the Goldsmiths Psychology Department's Ethics Board.

### 2.3. Overall materials

#### 2.3.1. Self-report measures

The self-report measures filled out at the end of each experimental session required participants to rate the familiarity with the passage in the exposure phase on a 7-point scale, their German speaking and writing abilities on 7-point scales, the amount of musical training they had received via the Training sub-scale of the Gold-MSI (Müllensiefen et al., 2014), as well as 14 questions assessing their affinity to Richard Wagner's music, each using a 5-point scale. In addition they also completed a 14-item objective multiple choice test assessing objective knowledge of *Der Ring des Nibelungen* and various facts relating to the life of Richard Wagner. Data from the Wagner affinity questionnaire was analyzed using factor analysis and each participant was assigned a corresponding factor score as described in Müllensiefen et al. (2016). Data from Wagner knowledge multiple choice test was scored using an item response model that generated an ability estimate for each participant (Müllensiefen et al., 2016).

#### 2.3.2. Measures of musical structure and sound

In order to assess each leitmotive's structural complexity, leitmotives were transcribed as a short monophonic melody into a symbolic music format and converted to a numerical tabular format suitable for melodic feature extraction using the FANTASTIC software toolbox (Müllensiefen, 2009). Four features that each capture a different aspect of melodic complexity and that had been used successfully to model cognitive behavior on melodic discrimination tests were extracted (see Müllensiefen, 2009; Harrison et al., 2016, for details): (1) Interval entropy, defined via the relative frequency of each melodic interval in the leitmotive, (2) Length, defined as the number of notes (3) Tonalness, defined as the highest of the 24 correlation coefficients as generated by the Krumhansl-Schmuckler key finding algorithm (Krumhansl, 2001). (4) Local step wise contour, defined as the mean absolute difference between adjacent values in the pitch contour vector of a melody.

These four features correlated highly across the 20 leitmotives, which suggested that they index a common dimension. Hence, principal component analysis (PCA) was used to aggregate the four features and derive a single measure of melodic complexity. The unidimensional PCA model using all four features explained 60% of the variance in the data, with Length having a relatively high uniqueness (0.59) value compared to the three other features (all values < 0.36). As a result, Length was removed and a unidimensional PCA model was run on the remaining 3 features which achieved to explain 70% of the variance in the data. PCA scores were derived from this model for each leitmotive and were used in the subsequent analysis to represent structural (i.e., melodic) complexity.

To assess audio similarity we used chromagram features (Mauch et al., 2015) that were extracted from the individual leitmotives on the recognition list. The audio data was extracted from the 10 min passage of the exposure phase of the experiment using Sonic-Annotator (Cannam et al., 2010). Chromagram features were then compared and the best alignment for each leitmotive within the 10 min passage was identified using database thresholding as implemented in the audioDB search engine (Rhodes et al., 2010).

### 2.4. Experiment I

#### 2.4.1. Design

The first experiment used a within-subjects design, with identical experimental conditions for all participants. The independent variables measured were musical training, German speaking skills, Wagner affinity, as well as objective Wagner knowledge. For each leitmotive, judgments of pleasantness, perceived conveyed valence, as well as arousal ratings were also taken to gather subjective assessments of the leitmotive stimuli for the models. Questions regarding the musical material were taken in real-time during the experiment in the order listed above and information regarding individual differences were taken after the listening portion of the experiment. Our item based model also incorporated a chroma measure that was indicative of how close the probed audio stimuli used in the experiment were to the audio used in the listening portion of the experiment. The dependent variable measured was whether or not a participant was able to correctly identify a leitmotive from a listening test, as well as the participant's subjective ratings of the musical material itself.

#### 2.4.2. Participants

For the first experiment a convenience sample (*N* = 100) was used, with additional effort made to recruit participants with either familiarity or affinity for the music of Richard Wagner from across the greater London area. The experiment was advertised over a host of mediums including posters, email lists, Twitter and general word-of-mouth to find individuals familiar with the music of Wagner. The sample was made up of 55 females (55%) and 45 males (45%) with a mean age of 28.7 (*R* = 18–65, *SD* = 11.82). Written consent was obtained from all participants and participants had the option of accepting £7 compensation for travel and time expenses.

#### 2.4.3. Materials and procedure

The musical stimuli of the first experiment were based off an earlier study by Albrecht and Frieler (2014). The scene was chosen for its narrative qualities and high concentration of leitmotive material. The audio used was taken from the second scene of the first act of *Siegfried* of the 1976 Pierre Boulez *Der Ring des Nibelungen* DVD recording at the Bayreuth Festspielhaus. This scene is colloquially referred to as the *Wanderer Scene*. Excerpts chosen for probes in the memory sequence were taken from the same Boulez recording.

Twenty probes containing the leitmotives were chosen after consulting the Burghold (1910) libretto as well as the Albrecht study. The 10 probes that occurred in the *Wanderer Scene* were chosen to mirror the initial Albrecht study, each occurring with various frequencies. The 10 probes used as lures were taken from a similar narrative passage from the same recording of *Götterdömmerung*. Leitmotives used as lures in the first experiment were consequently used as “targets” (i.e., leitmotives actually contained in the 10 min audio passage) in the second experiment. After the 20 leitmotives were chosen, renditions of each leitmotive were then taken from throughout the Boulez *Der Ring des Nibelungen* to serve as audio excerpts for the test phase. When possible, probes were chosen without simultaneous sounding vocals. Data was collected using a participant response sheet generated for the purpose of this experiment.

### 2.5. Experiment II

#### 2.5.1. Participants

The second experiment also used a convenience sample (*N* = 31) with additional effort made to recruit participants with specialized Wagner knowledge. The sample was made up of 16 females (52%) and 15 males (48%) with a mean age of 25.19 (*R* = 18–65, *SD* = 8.91). Participants from Experiment I were excluded from participating in Experiment II.

#### 2.5.2. Materials

Participants were played a 10 min excerpt prior to Siegfried's death scene from *Götterdämmerung*. The 20 leitmotives probes for the memory test were exactly the same as in Experiment II only that their assignment to targets (*old* items) and lures (*new* items) changed given the different passage in the exposure phase. While the number of leitmotive items labeled as *old* and *new* was split evenly in the first experiment, the constraint to use the same leitmotive items as for Experiment I, resulted in 13 items *old* and 7 *new* items for Experiment II.

## 3. Results

Across both samples the individual difference measures of Wagner knowledge and Wagner affinity were highly correlated (*r* = 0.71) and in order to avoid issues with multi-collinearity within the linear regression models used for analysis, both measures were subjected to a PCA which explained 85% of the variance with one dimension. Component factor scores for each participant were derived from the PCA model and were labeled as Wagner expertise.

Data modeling proceeded in three steps. The first model uses all data from both experiments and models participant responses only in terms of individual differences measures. The second model then uses significant individual difference measures identified in the first model and assesses whether the measure of structural leitmotive complexity as well as the number of occurrences of the leitmotive in the exposure phase contribute to modeling participant responses with *old* items. Here, we first assess data from Experiment I and Experiment II separately, and if model coefficients are comparable, we subsequently combine the data from both experiments. In the third step, we model responses to the *new* items including any significant individual differences measures as well melodic complexity in addition to sound similarity. All models use participants' binary responses as to whether a leitmotive was present or not in the 10 min passage during the exposure phase, scored either correct or incorrect, as the dependent variable. At all steps the data was modeled using generalized mixed effects models using participants as random effects and all models were fit using the “lme4” (Bates et al., 2015) package implemented in the statistical computing software “R” (R Core Team, 2013).

### 3.1. Model I: individual parameters

The data from all participants from Experiments 1 and 2 (*N* = 131) was used for the construction of Model I. Predictor variables initially specified for Model I were the Wagner expertise score, the musical training score from the Gold-MSI, and self-reported German speaking ability. In addition we used leitmotive as a second random effect in addition to participants to accommodate the fact that some leitmotive items might be generally more or less difficult. The initial model is given in Table 1 and shows that only Wagner expertise emerged as a significant predictor of leitmotive recognition ability, while neither the musical training score nor German speaking ability reached the common significance threshold of *p* < 0.05. As a result, only Wagner expertise was retained as a fixed effects predictor and the model was refit. The refit individual differences model had a predictive accuracy of 69.9% for the participant responses and showed a significantly (*p* < 0.001 on a likelihood ratio test) better fit to the data (BIC = 3,164) than a null model only including random effects for participants and leitmotives (BIC = 3,236). In addition, the fit was not significantly worse (*p* = 0.146) than the full model including all three individual differences measures (BIC = 3,176). Therefore, we only used Wagner expertise as an individual differences measure in the subsequent modeling stages.

**Table 1:** **Model I: individual differences variables**.

	**Coefficient**	**Standard Error**	***p*-value**
Intercept	0.61	0.20	<0.002^***^
Wagner Expertise	0.39	0.06	<0.001^***^
Musical Training	0.01	0.004	0.14
German Speaking Ability	−0.02	0.03	0.40

*** *p < 0.001*.

### 3.2. Model II: old items

For modeling responses to the old items two separate models were constructed for the data from Experiment I (*N* = 100) and II (*N* = 31). In addition to the random effect for participants and Wagner expertise as fixed predictor, number of occurrences of each leitmotive (as determined by the first author) in the exposure phase and the PCA scores for structural complexity were also included as fixed effects. Model parameters for both models were computed using the Laplace approximated maximum likelihood estimates and their 95% confidence intervals were determined by likelihood profiling. Parameter estimates and confidence intervals for both models are given in Table 2 which shows that for all three fixed effects parameters confidence intervals overlap substantially. Specifically, the parameter estimates for Model II are contained within the corresponding confidence intervals derived for Model I, indicating that the estimates derived from the two models are not significantly different from each other. After collapsing the data from both experiments we computed a full model including all main effects as well as interactions between the individual differences in Wagner expertise and the two experimental factors of times heard and structural complexity. This can be seen in Table 3. We then removed the non-significant interaction between times heard and Wagner expertise and obtained the final model as given in Table 3. When compared on the Bayesian Information Criterion fit index, this final model fit the data substantially better (BIC = 1,635) than a null model only including Wagner expertise (BIC = 1,675), a model only including main effects (BIC = 1,642) and a model including both interaction effects (BIC = 1,640). The final model had a predictive accuracy of 68.12% In line with with one of our hypotheses, Wagner expertise had a positive effect on memory performance, while structural melodic complexity had a negative effect. Not in line with our original hypotheses, the number of times a leitmotive occurred in the exposure phase had a negative effect on recognition rates. We provide a possible explanation for this finding below.

**Table 2:** **Model II: old items, Modeling item level data from experiment I and II separately**.

	**Experiment I**		**Experiment II**	
	**Coefficient**	**CI**	**Coefficient**	**CI**
Wagner Expertise	0.87	[0.17, 0.45]	0.57	[0.21, 0.95]
Times Heard	−0.03	[−0.04, −0.01]	−0.06	[−0.11, −0.02]
Structural Complexity	−0.39	[−0.52, −0.26]	−0.13	[−0.42, 0.14]

**Table 3:** **Model II: combining item level data from experiment I and II**.

	**Coefficient**	**Standard Error**	***p*-value**
Intercept	0.92	0.08	<0.001^***^
Wagner Expertise	0.38	0.07	<0.001^***^
Structural Complexity	−0.32	0.06	<0.001^***^
Times Heard	−0.03	0.01	<0.001^***^
Expertise Complexity Interaction	0.24	0.06	<0.001^***^

*** *p < 0.001*.

### 3.3. Model III: new items

For modeling the responses to the *new* items we followed the same modeling strategy of firstly modeling the data from Experiment I and consequently the data from Experiment II separately. Building on the results from steps 1 and 2, we included Wagner expertise as well as structural complexity as fixed effects predictors and added sound similarity based on the chromagram measure as a third predictor seen in Table 4. We did not include the number of times the leitmotive occurred in the exposure phase as a predictor because this variable has a constant value of zero for new items. After collapsing the data from both experiments we computed a full model including all main effects as well as interactions between the individual differences in Wagner expertise and the two experimental factors of structural complexity and chroma distance. We removed the non-significant interaction between chroma distance and Wagner expertise and obtained the final model as given in Table 5. When compared on the Bayesian Information Criterion fit index, this final model fit the data substantially better (BIC = 1,598) than a null model only including Wagner expertise (BIC = 1,624), a model including both interaction effects (BIC = 1,604) and was comparable to a model only have main effects (BIC = 1,597). The final mode had a predictive accuracy of 69.45%.

**Table 4:** **Model III: modeling of data for new items from experiment I and II**.

	**Experiment I Old Items**		**Experiment II Old Items**	
	**Coefficient**	**CI**	**Coefficient**	**CI**
Wagner Expertise	0.44	[0.27, 0.62]	0.40	[−0.04, 0.87]
Chroma Distance	1.04	[0.68, 1.42]	−1.86	[−4.93, 1.08]
Structural Complexity	0.40	[0.18, 0.62]	0.30	[0.06, 0.54]

**Table 5:** **Model III: combined data for new items**.

	**Coefficient**	**Standard Error**	***p*-value**
Intercept	−0.23	0.17	0.19
Wagner Expertise	0.39	0.08	<0.001^***^
Structural Complexity	0.35	0.08	<0.001^***^
Chroma Distance	0.71	0.13	<0.001^***^
Wager Expertise Complexity Interaction	0.23	0.09	<0.01^*^

* p < 0.05;

*** *p < 0.001*.

In line with our hypotheses, Wagner expertise has a positive effect on memory performance for new items, i.e., the ability to identify new items as not having heard before. Unlike its effect in the old item model, structural melodic complexity has a positive effect on correctly responding to new items with “not heard before” as does distance in terms of chromagram features.

## 4. Discussion

Consistent with our initial hypothesis, the results of both experiments demonstrate that these models of leitmotive memory performance are comprehensive in that they include both individual differences variables as well as symbolic and audio features of musical structure. Model I was able to reproduce results from previous work Müllensiefen et al. (2016) demonstrating that Wagner expertise was a significant predictor of a listener's memory for musical material. Of the three individual differences variables hypothesized to contribute to an individual's leitmotive recognition rate, only Wager expertise but not general musical training nor German speaking ability emerged as a significant predictor. One reason that musical training may not have emerged as a significant predictor in the individual differences model is that musical training and Wagner expertise are correlated. Using the mixed effects models it is not possible to model correlations between predictors and in this case the stronger predictor of Wagner expertise may be suppressing the weaker predictor of musical training, thus possibly explaining the different previous findings (Müllensiefen et al., 2016) due to a different modeling technique (structural equation modeling) that can handle correlated predictors. To our knowledge, this is one of the first analyses that has used a scaled measure of musical expertise other than musical training (i.e., stylistic expertise) which accounts for the largest amount of variance explained in a participant's response, though for an exception see Farrugia et al. (2016).

In addition to musical training not emerging as significant, German speaking abilities also did not reach significance, which might be attributed to either unintelligible diction from the Wagnerian singing that would not lead to more efficient encoding or from not having enough German speakers as a part of the sample. The results of the first model serve as initial evidence for a hypothesis assuming that there are more aspects of musical expertise that can be important for modeling music perception and cognition other than solely relying on musical training as an indicator for musical skills and expertise.

The second statistical model was able to confirm the hypothesis that measures of structural complexity of items in the test phase explain part of the variance in the participants' memory response data. This is consistent with other research using similar methodologies (Dewitt and Crowder, 1986; Croonen, 1994). More specifically, the second model demonstrated that the structural complexity of a leitmotive has a negative effect on an individual's ability to recognize musical material, while the amount of times heard surprisingly displayed a negative effect. The findings on structural complexity were not surprising in light of some literature with complexity serving as a predictor of memory recall (Harrison et al., 2016). The surprising finding of the negative relationship with times heard might be attributed to a variable not measured in this experiment that is related to perceptual salience.

In the passage used, the more perceptually salient motives occur less frequently than the others used in the excerpt. After re-examining the excerpt, we believe that the perceptually salient motives are those that are easier to detect and remember from the dense auditory scene. Those motives would be structurally simpler and in fact there is a clear negative correlation between our measures of complexity and the amount of times heard in the excerpt (*r* = −0.25), which means that simpler motives occur most often. In addition, the experimental design of the memory task introduced a correlation between structural length and complexity on one hand and the number of times that a motive was played in the test phase on the other hand, because shorter motives were repeated more often during the retrieval task. It is possible that these additional repetitions could also have facilitated memory retrieval. That said, measures of compositional complexity and simplicity are not all that contribute to perceptual saliency. Gestalt principles like *Prägnanz* or uniqueness with respect to a corpus are important as well. The aspect of uniqueness is connected to principles of statistical learning and can for example be measured by second order corpus features which have already proven to be powerful predictors in previous studies on melodic memory (Müllensiefen and Halpern, 2014). To follow up on this finding, future research will focus on investigating the extent to which compositional features reflecting perceptual salience or uniqueness can be used in respect to a large and appropriate corpus such as the Barlow and Morgenstern dictionary of operatic themes (Barlow and Morgenstern, 1966).

The third model aimed at explaining how listeners make memory decisions regarding musical material that they cannot recognize from a recent listening episode. It included measures of chroma distance and structural complexity as well as a significant interaction between Wagner expertise and structural complexity. Accounting for a small, yet significant proportion of the variance, the expertise and complexity interaction provides further evidence supporting the notion that listeners with different individual characteristics can react differently to the same musical stimulus features. In particular the interaction effect suggests an interpretation that listeners with high Wagner expertise benefit from the structural complexity of the leitmotives presented more strongly to make correct decisions about the novelty of the leitmotive item. Additionally Model III also includes a component that does not reflect compositional structure in a traditional music-theoretical sense, but rather deals directly with the sound itself. Interestingly the chroma distance variable exhibits the strongest effect among the predictors in the model (*b* = 0.71) and thus provides further evidence that measures that reflect properties of sound and the musical surface can make important contributions to models of music perception and cognition.

## 5. Conclusions

Overall, we believe the results from this experiment are able to help close the gap between experimental work that has relied heavily on artificial designs and musical stimuli for the sake of experimental control on one hand and research attempts to capture music listening in a more ecological setting on the other. The music of Richard Wagner has been notorious in its reputation for being difficult to comprehend, but the results from this study suggest that parsing the musical surface of something like *Der Ring des Nibelungen* is a process that is accomplished through repeated listening and active engagement with the music that does not require specialized musical training. Hearing these complex musical ideas is open to anyone and being able to hear salient musical events in a dense musical texture does not seem to be dependent on an individual's musical training. We believe that this is further evidence and reason for beginning to move closer to musical perception modeling that firstly moves away from using solely musical training as a proxy for musical ability and secondly incorporates recent work done in music informatics to help more accurately model perception.

## Ethics statement

This study was carried out in accordance with the recommendations of Goldsmiths Psychology Ethics Board, Goldsmiths Department of Psychology with written informed consent from all subjects. All subjects gave written informed consent in accordance with the Declaration of Helsinki. The protocol was approved by the Goldsmiths Psychology Ethics Board.

## Author contributions

DB- Experimental design, literature review, running of participants, draft manuscript, data cleaning. DM- Experimental design, data analysis, experimental guidance and overview.

## Funding

Funding for this research was provided by the AHRC Digital Transformations Transforming Musicology in the Digital Economy Programme AH/L006820/1.

### Conflict of interest statement

The authors declare that the research was conducted in the absence of any commercial or financial relationships that could be construed as a potential conflict of interest.
